# Supplementary material for: Performing tympanometry using smartphones
Source: Commun Med (Lond). 2022 Jun 16;2:57. doi: 10.1038/s43856-022-00120-9 (PMC9203539; doi:10.1038/s43856-022-00120-9)
Supplement: Supplementary file 2 — Description of additional supplementary files [file 43856_2022_120_MOESM2_ESM.pdf]

Description of Additional Supplementary Files

File Name: Supplementary Data 1
Description: Source Data

File Name: Supplementary Movie 1
Description: Video illustrating technique for testing.
