# Supplementary material for: Performing tympanometry using smartphones
Source: Commun Med (Lond). 2022 Jun 16;2:57. doi: 10.1038/s43856-022-00120-9 (PMC9203539; doi:10.1038/s43856-022-00120-9)
Supplement: Supplementary file 4 — Supplementary materials [file 43856_2022_120_MOESM4_ESM.pdf]

Performing tympanometry using smartphones

Justin Chan,[1*] Ali Najafi,[2] Mallory Baker,[3] Julie Kinsman,[3] Lisa R. Mancl,[4]
Susan Norton,[3,4,5] Randall Bly,[3,5*] Shyamnath Gollakota[1,2*]
[1]Paul G. Allen School of Computer Science and Engineering, University of Washington, Seattle, WA, USA
[2]Department of Electrical and Computer Engineering, University of Washington, Seattle, WA, USA
[3]Seattle Children's Hospital and Research Institute, Seattle, WA, USA
[4]Department of Speech & Hearing Sciences, University of Washington, Seattle, WA, USA
[5]Department of Otolaryngology — Head and Neck Surgery, University of Washington, Seattle, WA, USA
* Corresponding authors:
jucha@cs.washington.edu, randall.bly@seattlechildrens.org, gshyam@cs.washington.edu

Supplementary Note 1

Characterization of pressure leakage

We first characterize the amount of pressure leakage in a healthy adult ear without a history of middle ear disorders. To do this, we held the probe tip inside the ear to create a seal, decreased the pressure in the ear by -350 daPa, and measured how much negative pressure was lost after 20 s. We conducted this experiment for probe tip sizes ranging from 8 to 13 mm. Supplementary Table 2 shows that the rate of pressure leakage ranges from 2 to 4 daPa/s across all ear tip sizes, with a mean of 3 daPa/s. For reference, the pressure leakage when the probe tip is outside an ear and occluded with a piece of putty is 1 daPa/s.

In our clinical study across the 50 ears, the average pressure sweep duration and speed were 5 s and 125 daPa/s respectively. Across all ear tip sizes in this experiment, the amount of pressure leak over a 5 s measurement ranged from 10 to 20 daPa, with a mean of 13 daPa. The additional time to cover such a leak with a pressure speed of 125 daPa/s ranges from 0.1 to 0.2 s across all ear tip sizes, with a mean of 0.1 s.

Given a syringe plunger displacement of 5.3 mm needed to cover the full pressure range of 600 daPa (Figure 1d), and additional leaks, only 0.1 mm of that displacement would contribute to compensating for the pressure leak in our experiment. These results show that the amount of pressure leakage inherent to our device is minimal. We note that since our device uses the same ear tips as a commercial tympanometer, any pressure leaks that occur at the interface between the probe head and the ear canal, may also occur in a commercial tympanometer.

Effect of different syringe areas

We evaluated the accuracy of our system's volume estimates when measurements were performed in syringes of different areas. In this experiment, the system is calibrated to the cubic coefficients generated from measurements in a 5 mL syringe with a diameter of 12.5 mm and area of 123 mm^2 (Supplementary Figure 5). Measurements were then performed three times in a syringe of diameter 4.5 and 9 mm and area 16 and 64 mm^2 in increments of 1 mL. The syringes had a total measurable volume of 1 and 3 mL respectively. Prior work has shown that the cross-sectional area in the middle portion of the ear canal can range from 25 to 70 mm^2 (*1*). We compute the root-mean-square error of the calibrated tympanograms across the full pressure range of -400 to 200 daPa (Supplementary Table 6). Across all measured volumes in the syringes of area 16 mm and 64 mm^2, the average root-mean-square error was 0.04 ± 0.00 and 0.06 ± 0.02 mL respectively. This is comparable to the syringe of area 123 mm^2 which had an average root-mean-square error of 0.09 ± 0.02 mL.

Test-retest reliability

We evaluated the test-retest reliability of our smartphone-based system and a commercial tympanometer used in the clinical study (Supplementary Table. 5). Each device was tested on a single healthy

adult ear five consecutive times with the ear probe removed between each measurement. The pressure speed of the smartphone device was on average 186 ± 7 daPa/s across all five measurements. The pressure speed of the commercial device was set to 200 daPa/s. Peak admittance for the smartphone and GSI TympStar Pro ranged from 0.60 to 0.71 mL and 0.59 to 0.73 mL respectively, showing similar levels of test-retest variability. Ear canal volume ranged from 0.88 to 1.15 mL and 1.10 to 1.40 mL, and peak pressure ranged from -35 to -10 daPa and -4 to 0 daPa for the smartphone and commercial device respectively.

Effect of concurrent Bluetooth connections

In this experiment, we evaluated the effect of multiple nearby Bluetooth connections when performing the calibration procedure in Supplementary Figure 5. For this setup, we had one smartphone streaming audio over Bluetooth to a pair of headphones. We then had a second smartphone act as a Bluetooth hotspot that streamed a video over the air to a third smartphone. Finally, the smartphone connected to the tympanometer was concurrently playing a video while measurements were recorded. In this setup, the average root-mean-square error of the calibrated tympanograms was 0.09 ± 0.06 mL across all volumes. This is comparable to the error of 0.09 ± 0.02 mL when the calibration procedure was performed without nearby concurrent Bluetooth connections.

Testing at 1000 Hz

Our system can be configured to emit different frequency tones in software including at 1000 Hz. The 1000 Hz tone was calibrated to transmit at 85 dB SPL, which is the same sound level output as used for the 226 Hz tone in our clinical study. We measured the tympanogram at 1000 Hz in a single healthy adult ear without prior middle ear history in Supplementary Figure 12. We note that further testing is required to evaluate the utility of our system in young infants.

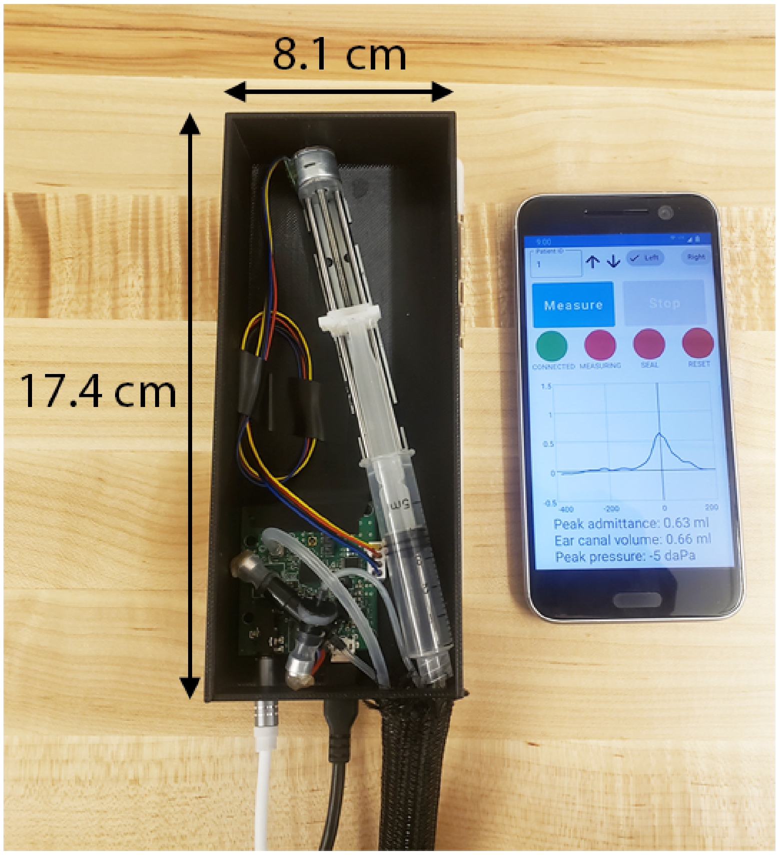

Supplementary Figure 1: **Interior of portable tympanometer.**

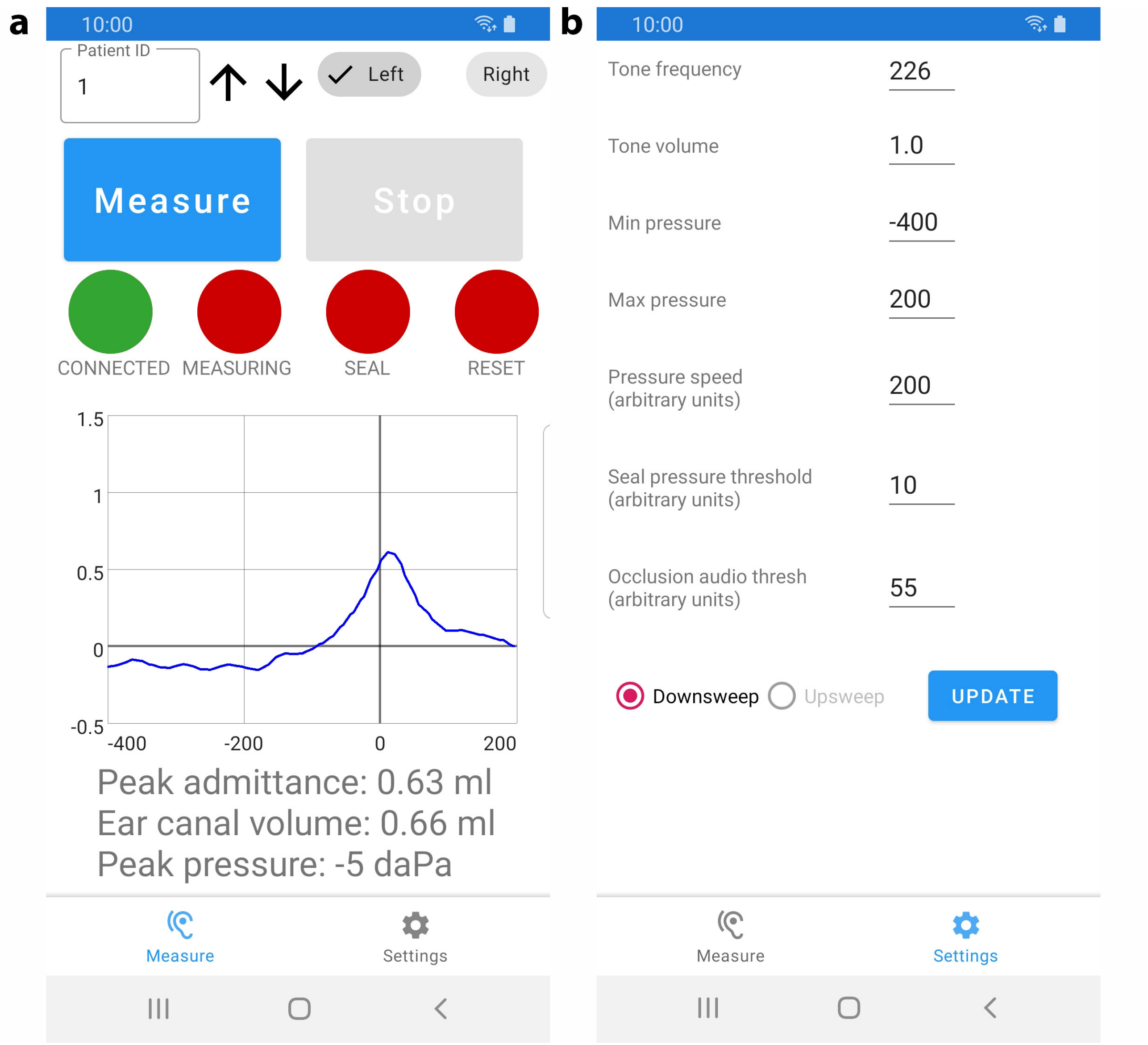

Supplementary Figure 2: **User interface of smartphone tympanometery software. a** Measurement screen shows measured tympanogram and clinical measures. Status indicators provide feedback about the measurement. **b** List of tympanometer settings that can be modified.

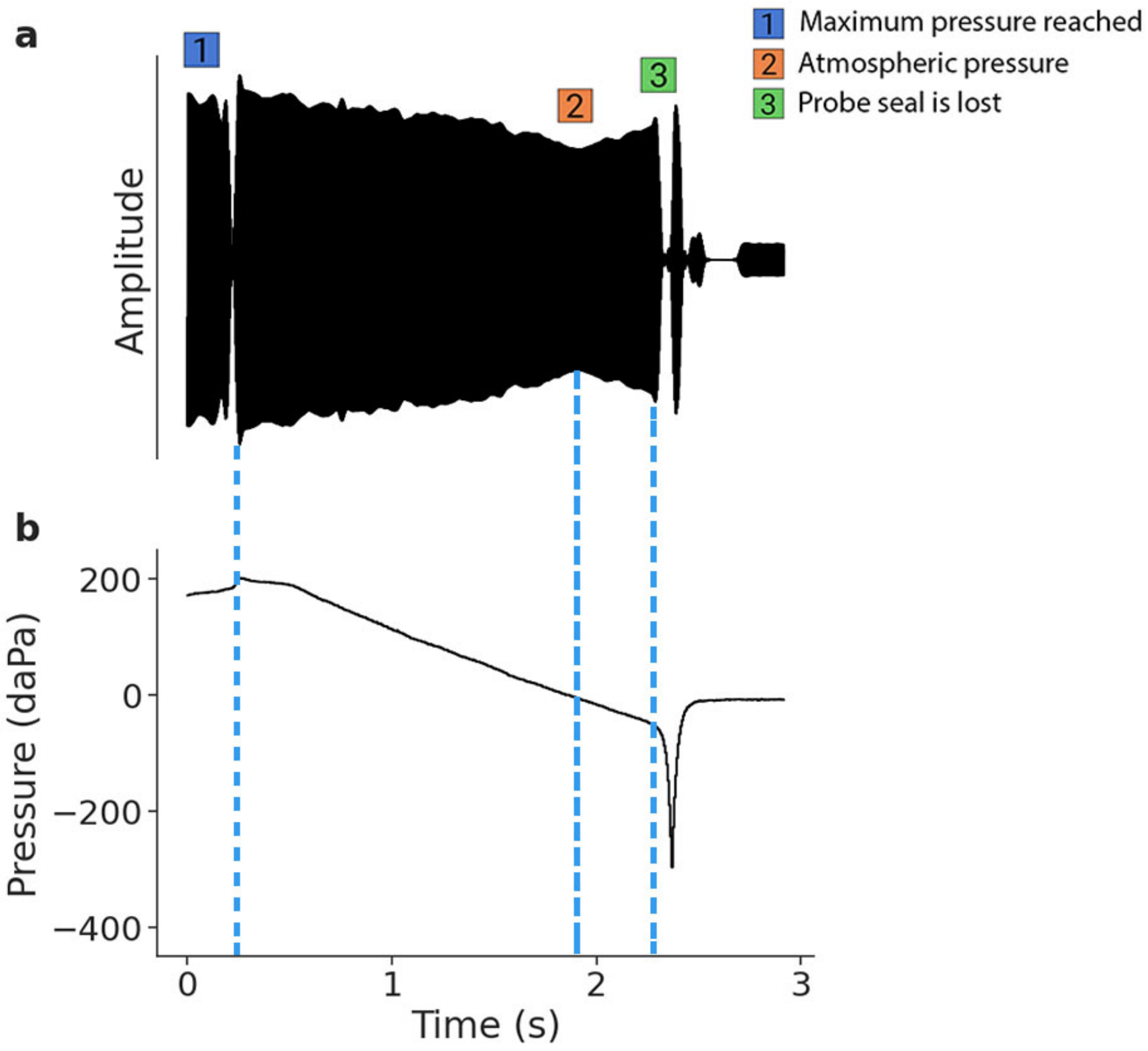

Supplementary Figure 3: **Acoustic and pressure signals when a seal is lost.** **a** When the seal is lost and the probe is dislodged, there is significant interference in the acoustic signal followed by a decrease in amplitude. **b** The pressure signal spikes downwards as the seal is lost, and quickly returns to atmospheric pressure when the probe tip is out of the ear.

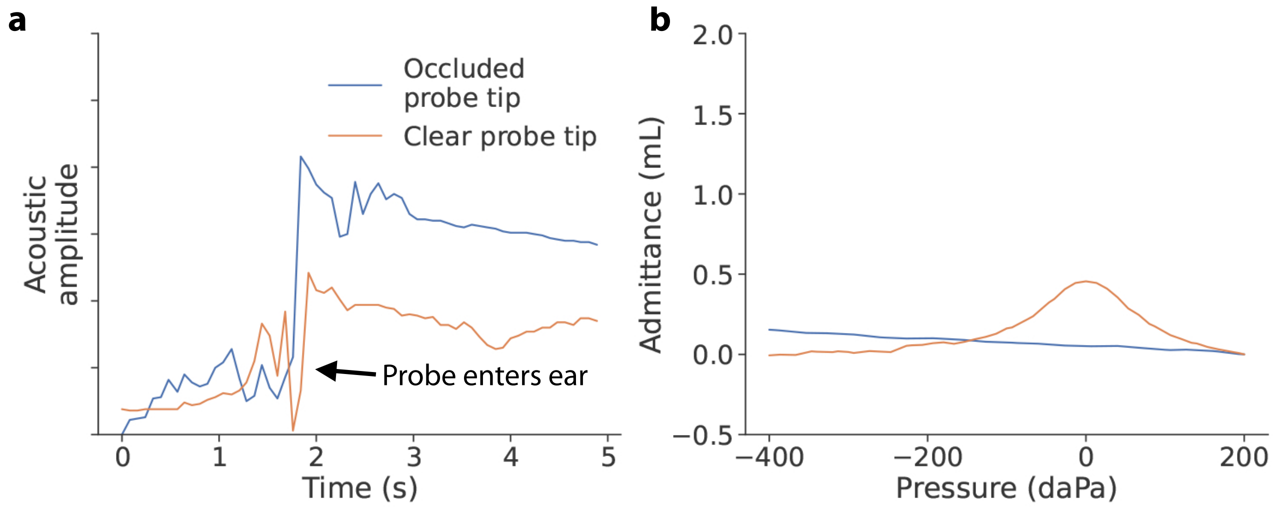

Supplementary Figure 4: **Occluded probe tip.** **a** The amplitude of the reflected acoustic signal is higher when the probe tip is occluded, compared to when it is clear. **b** The tympanograms produced in a normal and healthy adult ear with a clear probe tip and an occluded probe tip.

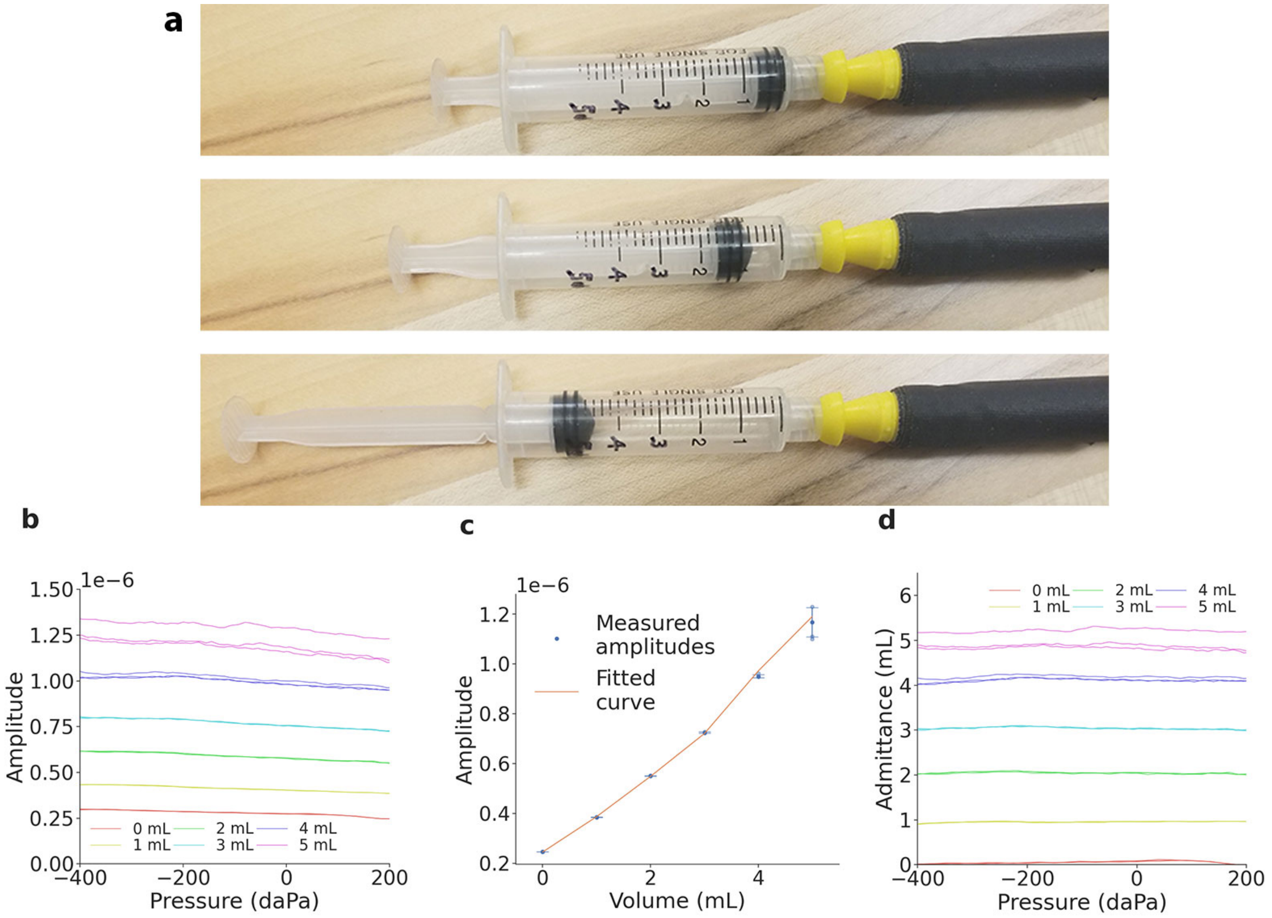

e

Volume (mL)	Root-mean-square error from -400 to 200 daPa (mL)
0	0.06 ± 0.01
1	0.05 ± 0.00
2	0.05 ± 0.01
3	0.05 ± 0.00
4	0.14 ± 0.04
5	0.18 ± 0.05
Mean	0.09 ± 0.02

Supplementary Figure 5: **Calibration procedure.** **a** Calibrating device in a 5 mL syringe in 1 mL increments. The figure shows the measurement for 0, 1, and 5 mL. **b** Uncalibrated tympanograms from different hard-backed volumes. **c** Measured acoustic amplitudes at different volumes. The points and errors bars indicate the mean and SD across three measurements. **d** Calibrated tympanograms in different volumes. **e** Root-mean-square error of calibrated tympanograms for different volumes.

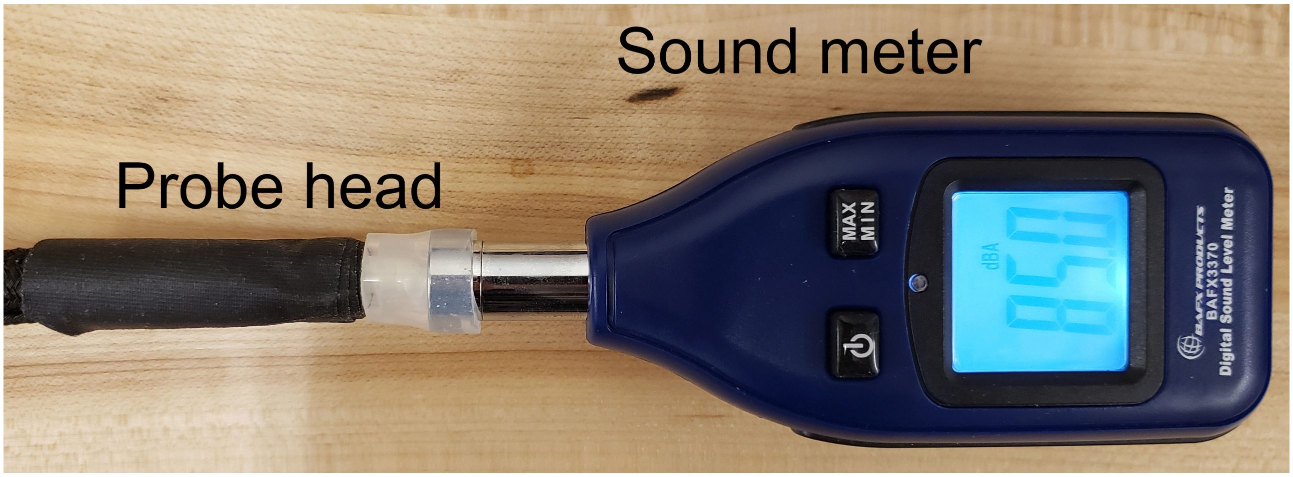

Supplementary Figure 6: **Sound level calibration.** A sound meter (BAFX 3370, Digital Sound Level Meter, $18) is used to calibrate the sound level produced by our system.

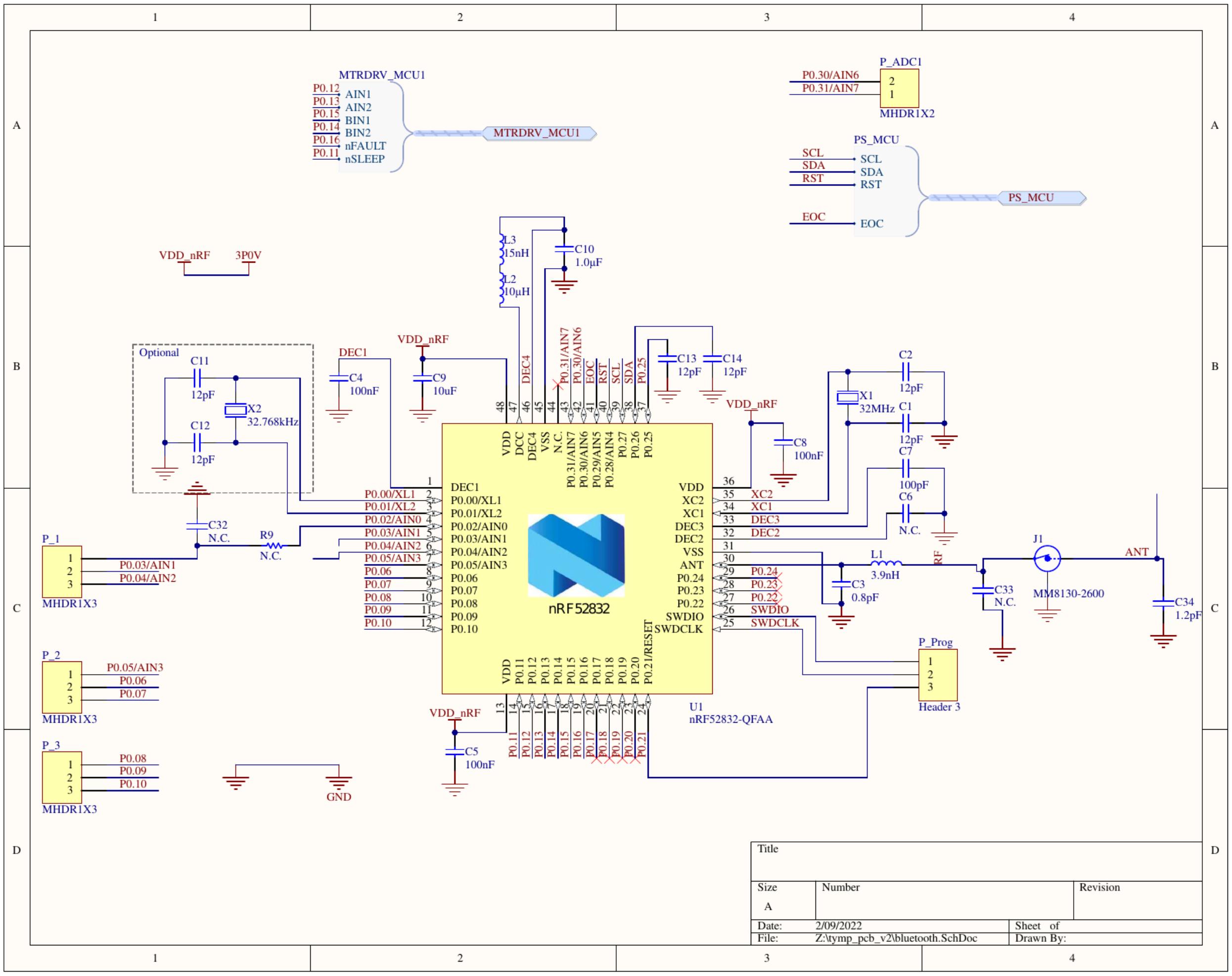

Supplementary Figure 7: **Schematic of microcontroller connections.**

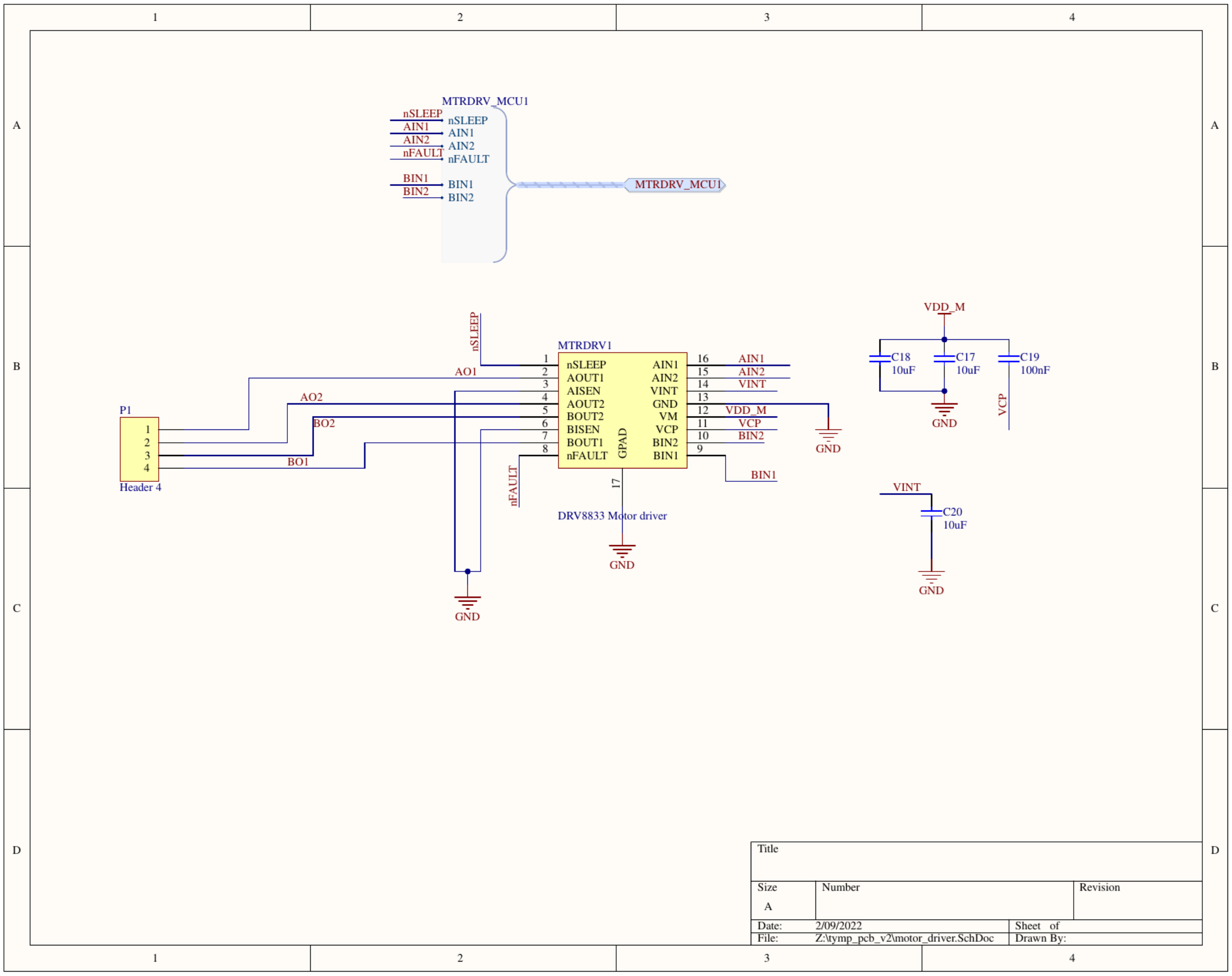

Supplementary Figure 8: **Schematic of motor driver connections.**

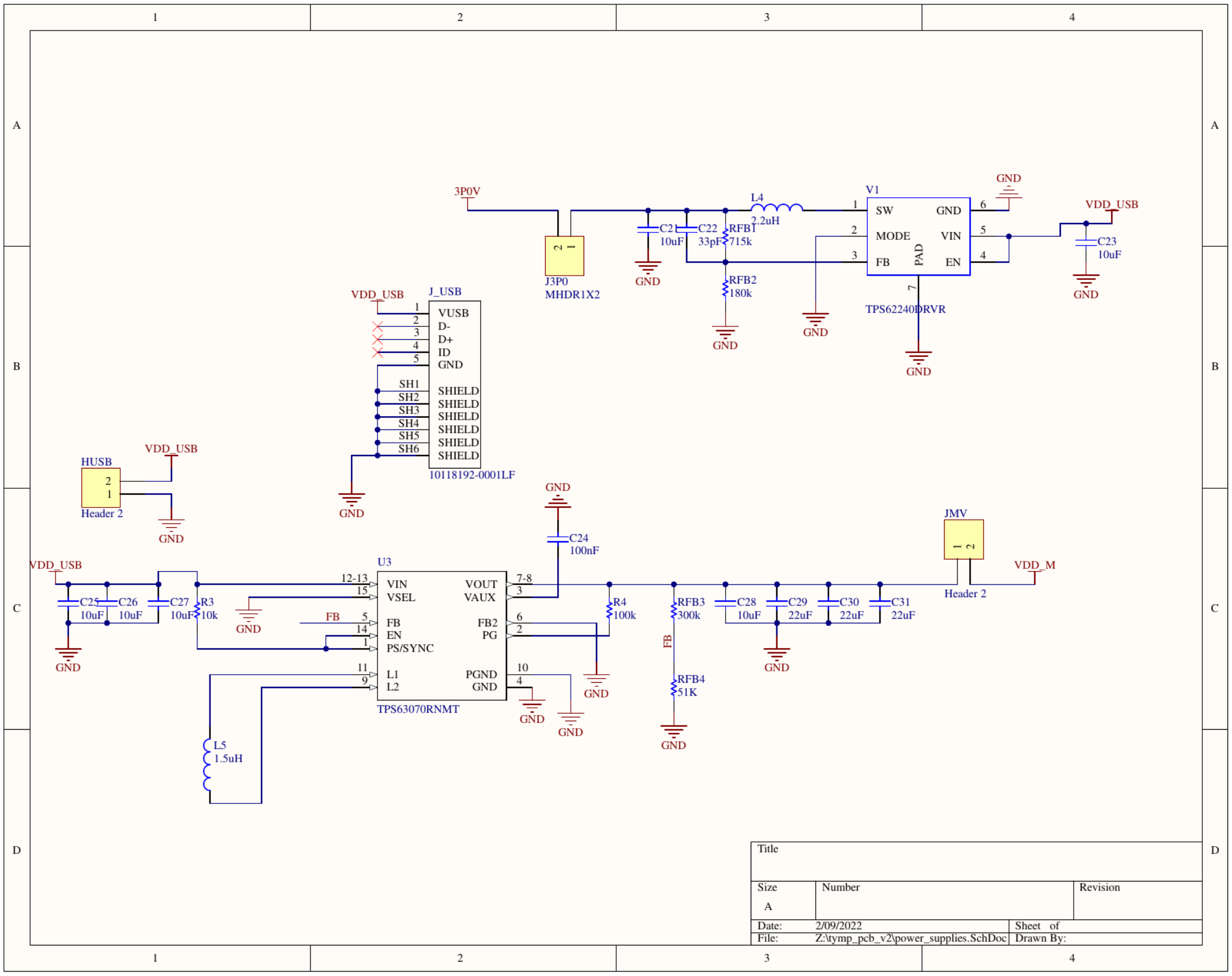

Supplementary Figure 9: **Schematic of power supply connections.**

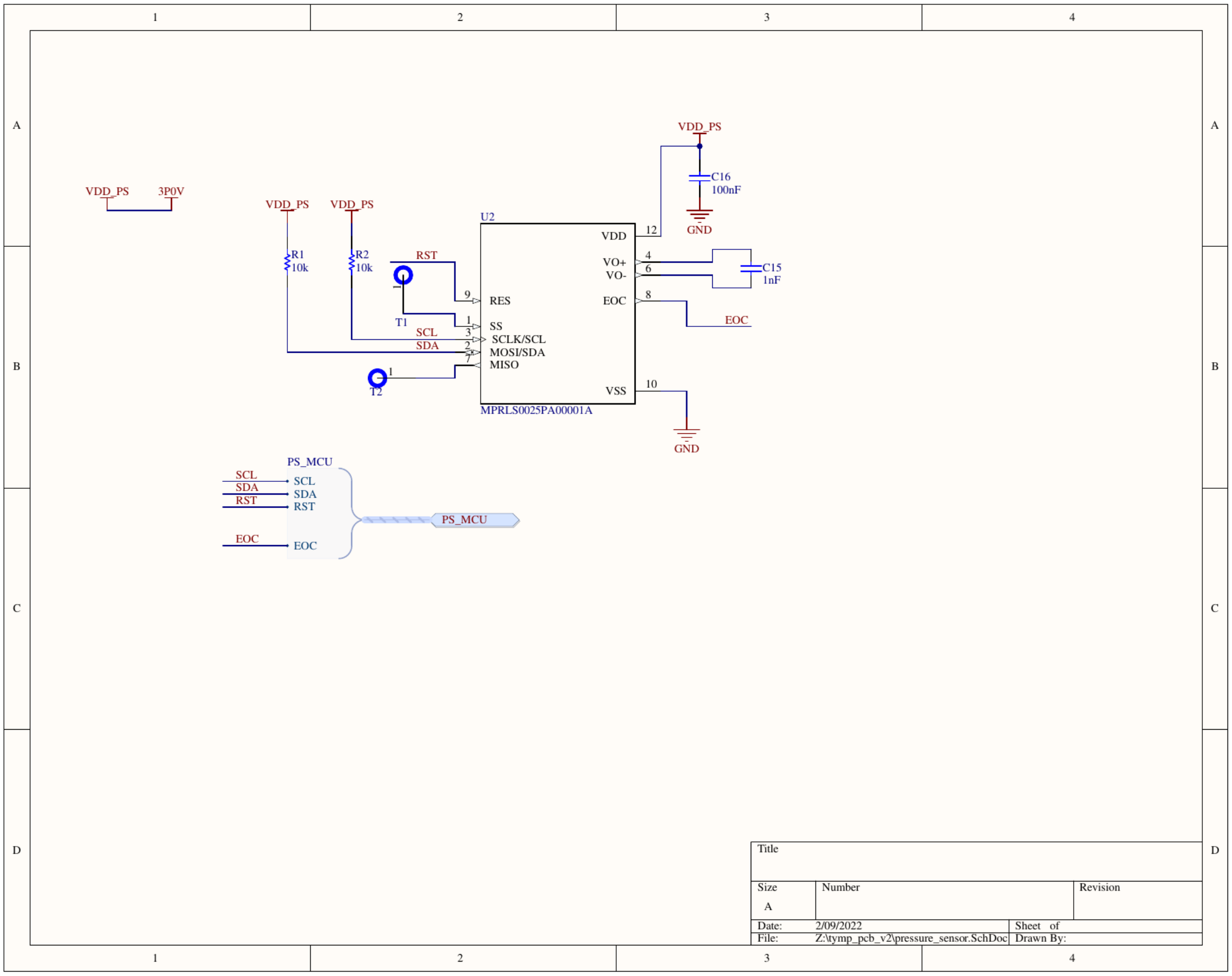

Supplementary Figure 10: **Schematic of pressure sensor connections.**

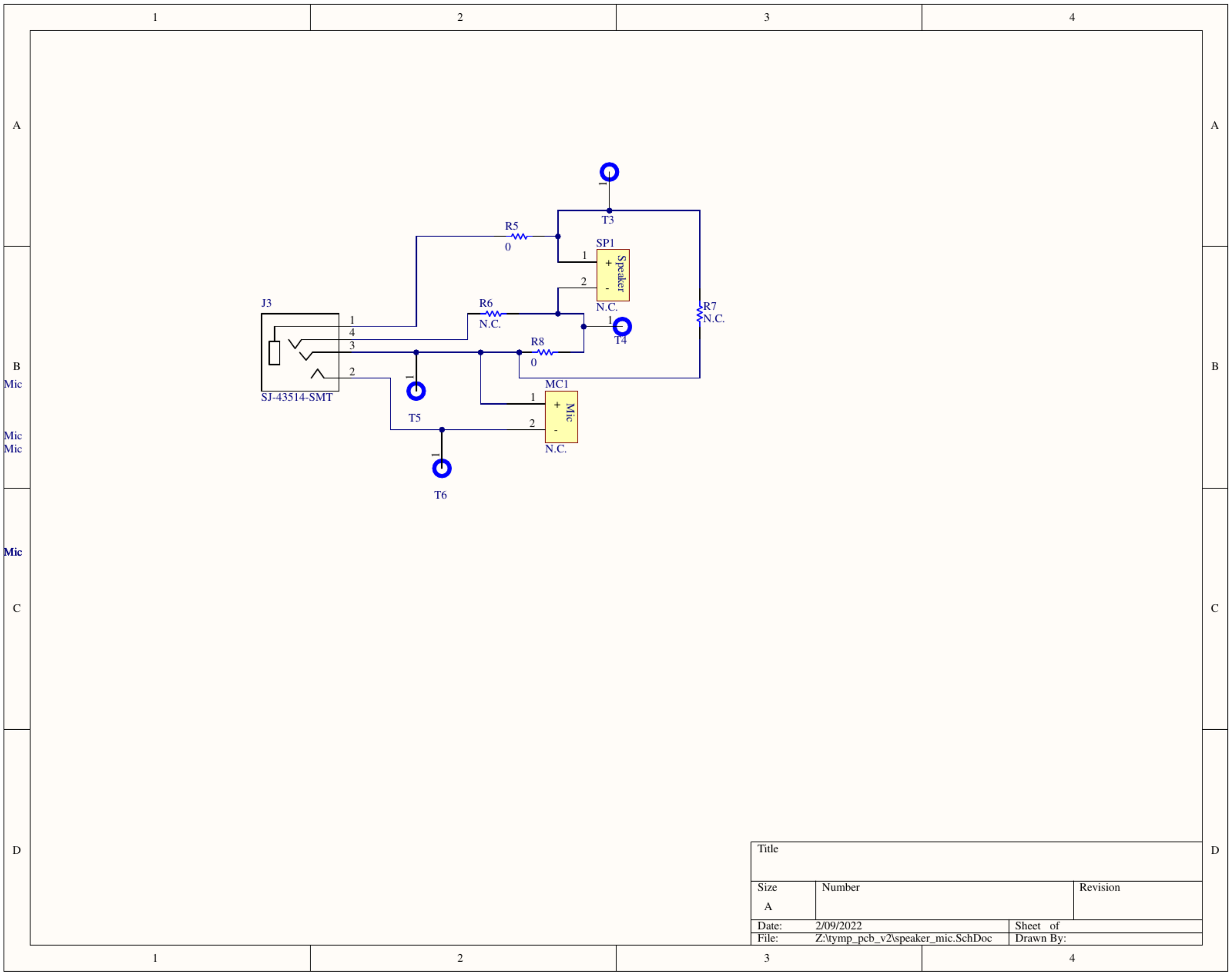

Supplementary Figure 11: **Schematic of speaker and microphone connections.**

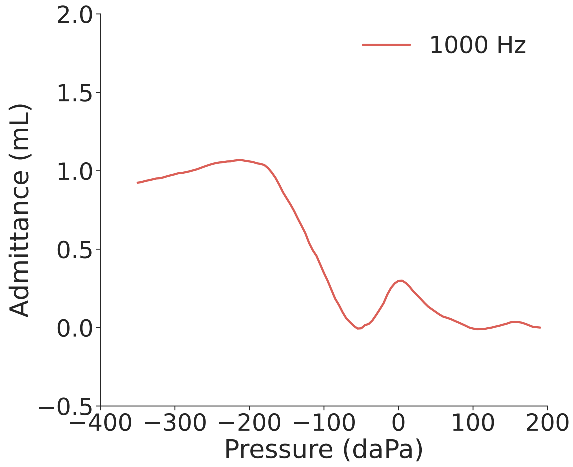

Supplementary Figure 12: **Benchmark testing at 1000 Hz in a healthy adult ear.**

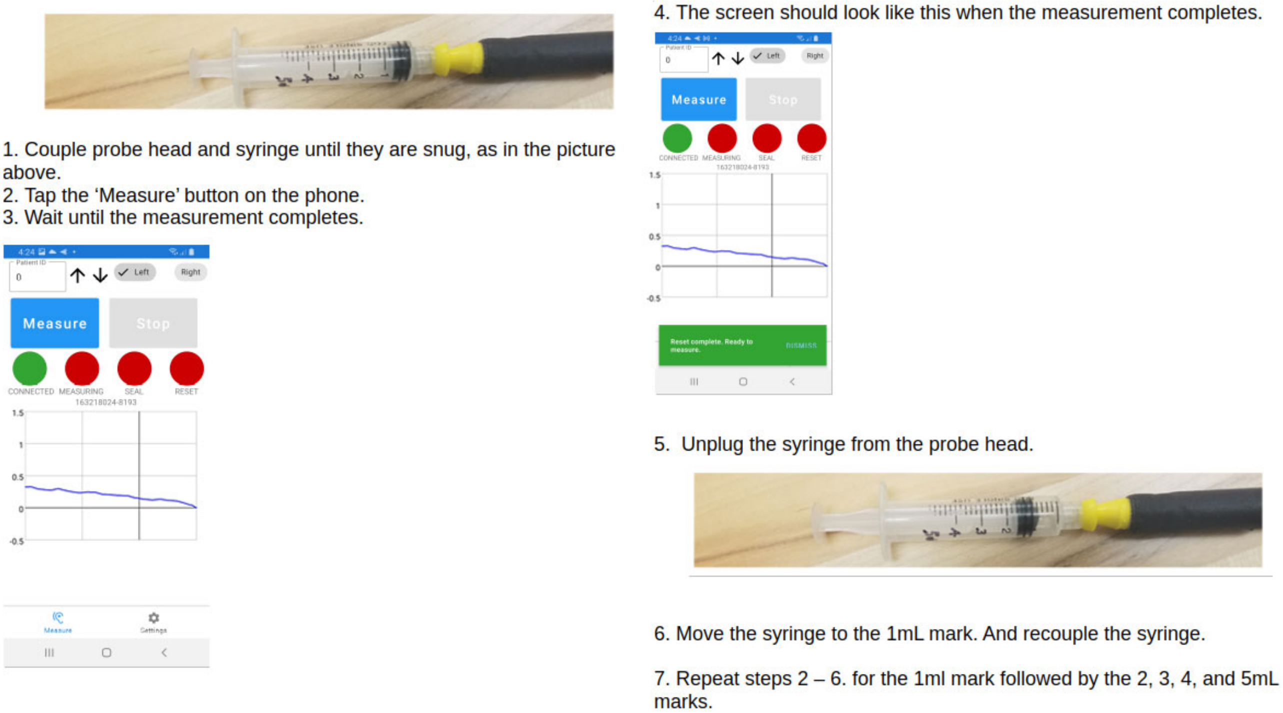

Supplementary Figure 13: **Instructions to calibrate device.**

Component	Cost
**Electronics**	
Speaker<br>(Knowles SR-32453-000)	$4.42
Microphone<br>(DB Unlimited MO064404-1)	$0.29
Stepper motor<br>(DC 2-Phase 4-wire Stepper Motor)	$6.16
Motor driver<br>(Toshiba TB6612FNG)	$0.90
Pressure sensor<br>(Honeywell MPRLS0025PA00001A)	$3.97
Microcontroller<br>(Nordic Semiconductor nRF52832)	$2.28
**Passive components**	
Syringe<br>(Frieda, 5 ml)	$0.55
Silicone tubing<br>3× ID: 1 mm, OD: 2 mm, Length: 1 m<br>1× ID: 2 mm, OD: 3 mm, Length: 80 mm<br>2× ID: 3 mm, OD: 4 mm, Length: 20 mm	$3.72
Y-connector<br>(Cole-Parmer, 1/8" × 1/8" × 1/8")	$0.63
Probe head<br>(Cole-Parmer Male luer with Lock Ring, 3/16")	$0.67
Tubing protector<br>(1/2" diameter, 1 m length)	$0.13
**Cabling**	
3.5 mm male-to-male audio cable	$0.29
Micro-USB power cable	$0.55
**PCB**	
Fabrication	$0.61
PCB assembly	$1.86
Final assembly	$1.41
**Total**	$28.44

Supplementary Table 1: **Raw material cost of components**. Costs for components, PCB fabrication, and assembly, for 1000 units.

	Ear tip size (mm)	Pressure leak (daPa/s)	Pressure leak over 5s measurement (daPa)	Time to cover leak with a pressure speed of 125 daPa/s (s)	Syringe displacement (mm)
	8	3	15	0.1	0.1
	9	3	15	0.1	0.1
	10	2	10	0.1	0.1
	11	4	20	0.2	0.2
	12	2	10	0.1	0.1
	13	2	10	0.1	0.1
**Mean values**	-	3	13	0.1	0.1

Supplementary Table 2: **Characterization of pressure leakage.**

**Age (years)**	9 ± 5
**Sex**	
Male, *n* (%)	33 (66)
Female, *n* (%)	17 (34)
**History of otitis media**	
Yes, *n* (%)	12 (24)
No, *n* (%)	38 (76)
**Cerumen obstruction**	
Yes, *n* (%)	9 (18)
No, *n* (%)	41 (82)
**Hearing loss**	
Conductive, *n* (%)	9 (18)
Sensorineural, *n* (%)	19 (38)
None, *n* (%)	23 (46)
Ear tip diameter (mm)	11 ± 1

Supplementary Table 3: **Demographic summary of clinical study participants.**

	Adults (11 years and up)	Children (6 months – 10 years)
**Static compliance**	0.3 to 1.4 mL (mean 0.8)	0.2 to 0.9 mL (mean 0.5)
**Ear canal volume:**	0.6 to 1.5 mL	0.3 to 0.9 mL
**Middle ear pressure:**	-110 to 150 daPa	-150 to 150 daPa

Tympanometry types: (Liden and Jerger classifications)

1. **Type A:** normal "A or mountain" shape
2. **Type B:** flat tracing non-measurable static compliance
3. **Type C:** negative middle ear air pressure
   (a) Children 6 months to 10 years: peak pressure less than -150 daPa
   (b) 11 years of age and up: peak pressure than -110 daPa
4. **Type As:** height is decreased or shallow (i.e. otosclerosis or fluid), compliance 0.2 mL or 0.1 mL
5. **Type Ad:** height is significantly increased of deep (i.e. ossicular disarticulation)
   (a) Children 6 months to 10 years: compliance 1.0 mL or greater
   (b) 11 years of age and up: compliance 1.5 mL of greater

Supplementary Table 4: **Clinical criteria for tympanogram classification.** Criteria and normative tympanometry values used by audiologists at our institution for classifying tympanograms into Liden and Jerger classifications (*2*–*5*).

	Smartphone system	GSI TympStar Pro
Peak admittance (mL)	0.60 – 0.71	0.59 – 0.73
Ear canal volume (mL)	0.88 – 1.15	1.10 – 1.40
Peak pressure (daPa)	-35 – -10	-4 – 0

Supplementary Table 5: **Test-retest reliability between smartphone and commercial tympanometer.**

Syringe area: 16 mm^2

Volume (mL)	Root-mean-square error from -400 to 200 daPa (mL)
0	0.03 ± 0.01
1	0.04 ± 0.01
Mean	0.04 ± 0.00

Syringe area: 64 mm^2

Volume (mL)	Root-mean-square error from -400 to 200 daPa (mL)
0	0.04 ± 0.01
1	0.06 ± 0.02
2	0.05 ± 0.03
3	0.09 ± 0.02
Mean	0.06 ± 0.02

Supplementary Table 6: **Effect of different syringe areas.** Root-mean-square error of calibrated tympanograms for syringes of area 16 and 64 mm^2 with a total measurable volume of 1 and 3 mL respectively.

Tympanogram type	Mean and standard deviation of absolute error (mL)	Mean and standard deviation of the differences (mL)
Type A	0.10 ± 0.06	0.02 ± 0.11
Type Ad	0.52 ± 0.00	-0.52 ± 0.00
Type As	0.18 ± 0.06	0.18 ± ± 0.06
Type C	0.01 ± 0.00	0.01 ± 0.00

Supplementary Table 7: **Tympanogram peak admittance error.**

Tympanogram type	Root-mean-square error (mL)
Type A	0.17 ± 0.11
Type Ad	0.34 ± 0.00
Type As	0.11 ± 0.02
Type B	1.13 ± 0.75
Type C	0.03 ± 0.00

Supplementary Table 8: **Root-mean-square error between tympanograms measured on the smartphone and commercial tympanometer.**

Tympanogram type	Mean and standard deviation of absolute error (mL)	Mean and standard deviation of the differences (mL)
Type A	0.20 ± 0.19	0.10 ± 0.26
Type Ad	0.26 ± 0.00	0.26 ± 0.00
Type As	0.10 ± 0.03	0.01 ± 0.10
Type B	2.35 ± 1.78	-2.35 ± 1.78
Type C	0.05 ± 0.00	0.05 ± 0.00

Supplementary Table 9: **Tympanogram ear canal volume error.**

Tympanogram type	Mean and standard deviation of absolute error (daPa)	Mean and standard deviation of the differences (daPa)
Type A	13 ± 11	2 ± 17
Type Ad	20 ± 0	-20 ± 0
Type As	13 ± 9	-3 ± 15
Type C	3 ± 0	3 ± 0

Supplementary Table 10: **Tympanogram peak pressure error.**

Algorithm 1: Algorithm to synchronize acoustic and pressure signals

Function `synchronize_signals` (*sig,pvals*) **:**

Input : sig acoustic signal from microphone
Input : $pvals$ pressure signal from microcontroller
Input : $maxtime$ timestamp of Bluetooth notification when pressure has reached 200 daPa
Input : $mintime$ timestamp of Bluetooth notification when pressure has reached -400 daPa
Output: sig acoustic signal clipped to start and end of pressure sweep
Output: $pvals$ pressure signal clipped to start and end of pressure sweep

// bandpass signal to [220,230] Hz, and obtain envelope
$filt \leftarrow bandpass(sig, [220, 230])$
$env \leftarrow envelope(filt)$

// find point in sig where pressure has peaked to 200 daPa
$min\_idx \leftarrow argmin(env[maxtime - 0.5s : maxtime])$
$min\_idx \leftarrow min\_idx + (maxtime - 0.5s)$
$max\_idx \leftarrow argmax(env[min\_idx : maxtime])$
$start\_point \leftarrow max\_idx + min\_idx$

// find point sig where pressure has dipped to -400 daPa
$min\_idx \leftarrow argmin(env[mintime - 0.5s : mintime])$
$min\_idx \leftarrow min\_idx + (mintime - 0.5s)$
$max\_idx \leftarrow argmax(env[mintime - 0.5s : min\_idx])$
$end\_point \leftarrow max\_idx + (mintime - 0.5s)$

// clip acoustic and pressure signals to timepoints when the pressure sweep from 200 to -400 daPa occurs
$sig = sig[start\_point : end\_point]$
$pvals = pvals[argmax(pvals) : argmax(pvals)]$

return $sig, pvals$

Supplementary References

1. Stinson, M. R. & Lawton, B. Specification of the geometry of the human ear canal for the prediction of sound-pressure level distribution. *The Journal of the Acoustical Society of America* **85**, 2492–2503 (1989).

2. Liden, G., Peterson, J. & Bjorkman, G. Tympanometry a method for analysis of middle—ear function. *Acta Oto-Laryngologica* **69**, 218–224 (1970).

3. Jerger, J. Clinical experience with impedance audiometry. *Archives of otolaryngology* **92**, 311–324 (1970).

4. Liden, G., Harford, E. & Hallen, O. Automatic tympanometry in clinical practice. *Audiology* **13**, 126–139 (1974).

5. Lidén, G., Harford, E. & Hallén, O. Tympanometry for the diagnosis of ossicular disruption. *Archives of Otolaryngology* **99**, 23–29 (1974).
