# Supplementary material for: Performing tympanometry using smartphones
Source: Commun Med (Lond). 2022 Jun 16;2:57. doi: 10.1038/s43856-022-00120-9 (PMC9203539; doi:10.1038/s43856-022-00120-9)
Supplement: Supplementary file 6 — Reporting Summary [file 43856_2022_120_MOESM6_ESM.pdf]

nature research

Corresponding author(s): Justin Chan, Randall Bly, Shyamnath Gollakota

Last updated by author(s): Apr 25, 2022

Reporting Summary

Nature Research wishes to improve the reproducibility of the work that we publish. This form provides structure for consistency and transparency in reporting. For further information on Nature Research policies, see our Editorial Policies and the Editorial Policy Checklist.

Statistics

For all statistical analyses, confirm that the following items are present in the figure legend, table legend, main text, or Methods section.

n/a	Confirmed	
☐	☒	The exact sample size (n) for each experimental group/condition, given as a discrete number and unit of measurement
☐	☒	A statement on whether measurements were taken from distinct samples or whether the same sample was measured repeatedly
☒	☐	The statistical test(s) used AND whether they are one- or two-sided<br>*Only common tests should be described solely by name; describe more complex techniques in the Methods section.*
☒	☐	A description of all covariates tested
☒	☐	A description of any assumptions or corrections, such as tests of normality and adjustment for multiple comparisons
☐	☒	A full description of the statistical parameters including central tendency (e.g. means) or other basic estimates (e.g. regression coefficient) AND variation (e.g. standard deviation) or associated estimates of uncertainty (e.g. confidence intervals)
☒	☐	For null hypothesis testing, the test statistic (e.g. F, t, r) with confidence intervals, effect sizes, degrees of freedom and P value noted<br>*Give P values as exact values whenever suitable.*
☒	☐	For Bayesian analysis, information on the choice of priors and Markov chain Monte Carlo settings
☒	☐	For hierarchical and complex designs, identification of the appropriate level for tests and full reporting of outcomes
☐	☒	Estimates of effect sizes (e.g. Cohen's d, Pearson's r), indicating how they were calculated

Our web collection on statistics for biologists contains articles on many of the points above.

Software and code

Policy information about availability of computer code

Data collection	Custom code was written for the Android smartphone and microcontroller to collect tympanometry data for the study.
Data analysis	Custom MATLAB scripts were used to analyze the data. The following software was used:<br>Matlab R2021a

For manuscripts utilizing custom algorithms or software that are central to the research but not yet described in published literature, software must be made available to editors and reviewers. We strongly encourage code deposition in a community repository (e.g. GitHub). See the Nature Research guidelines for submitting code & software for further information.

Data

Policy information about availability of data

All manuscripts must include a data availability statement. This statement should provide the following information, where applicable:

- Accession codes, unique identifiers, or web links for publicly available datasets
- A list of figures that have associated raw data
- A description of any restrictions on data availability

All data supporting the findings from this study are available within the article and its supplementary information. Source data are provided as Supplementary Data 1 with this paper.

Field-specific reporting

Please select the one below that is the best fit for your research. If you are not sure, read the appropriate sections before making your selection.

☒ Life sciences ☐ Behavioural & social sciences ☐ Ecological, evolutionary & environmental sciences

For a reference copy of the document with all sections, see nature.com/documents/nr-reporting-summary-flat.pdf

Life sciences study design

All studies must disclose on these points even when the disclosure is negative.

Sample size	Sample sizes were chosen based on whether sufficiently representative data was collected.
Data exclusions	No data was excluded.
Replication	The findings can be replicated.<br>Each ear in the clinical study was measured once each on the smartphone and the commercial device.
Randomization	Randomization of patients is not relevant. Patients were not allocated into different groups.
Blinding	Investigators were not blinded.

Reporting for specific materials, systems and methods

We require information from authors about some types of materials, experimental systems and methods used in many studies. Here, indicate whether each material, system or method listed is relevant to your study. If you are not sure if a list item applies to your research, read the appropriate section before selecting a response.

Materials & experimental systems

n/a	Involved in the study	
☒	☐	Antibodies
☒	☐	Eukaryotic cell lines
☒	☐	Palaeontology and archaeology
☒	☐	Animals and other organisms
☐	☒	Human research participants
☒	☐	Clinical data
☒	☐	Dual use research of concern

Methods

n/a	Involved in the study	
☒	☐	ChIP-seq
☒	☐	Flow cytometry
☒	☐	MRI-based neuroimaging

Human research participants

Policy information about studies involving human research participants

Population characteristics	The age range of the patients was 1 to 20 years with a mean age of 9 ± 5 years, and a female-to-male ratio of 0.52. All patients in the study were scheduled for an appointment with an audiologist and were undergoing tympanometry as part of their clinical visit.
Recruitment	Parental permission was obtained for participants under the age of 18 years. Children age 7 to 17 provided written assent. Assent was obtained after parental permission was granted. Children age 7 to 12 signed a simple assent form and children age 13 to 17 signed a consent form. Parents co-signed the consent form. Participants 18 years and older signed a consent form. Patients who were undergoing tympanometry as part of their clinical visit were recruited during routine audiology appointments.
Ethics oversight	Seattle Children's Hospital Institutional Review Board (STUDY00002820)

Note that full information on the approval of the study protocol must also be provided in the manuscript.
